# Women’s independent decision-making power and determinants on not to use contraceptives among currently married women in Ethiopia using demographic and Health Survey data: Multilevel Analysis

**DOI:** 10.1186/s12905-022-02051-y

**Published:** 2022-12-22

**Authors:** Desalegn Tesfa, Melkalem Mamuye Azanaw, Alemayehu Digssie Gebremariam, Melaku Tadege Engidaw, Mulu Tiruneh, Melkamu Aderajew Zemene, Denekew Tenaw Anley, Anteneh Mengist Dessie

**Affiliations:** Department of Public Health, College of Health Sciences, Debre Tabor University, Debre Tabor, Ethiopia

**Keywords:** Independent decision making, Not to use a contraceptive, Currently married women

## Abstract

**Background:**

Evidence suggests that couples frequently dispute regarding the desirability of pregnancy, as well as whether or not to employ family planning measures. There are numerous unmet needs owing to partner or family objections, according to a scares study that illustrates women’s independent decision-making capacity on whether or not to use a contraceptive. As a result, the purpose of this study was to analyze women’s independent decision-making power and determinants of not using contraceptives.

**Methods:**

Reproductive age group women aged (15–49 years) currently married who are not pregnant and are currently not using family planning preceding five years the survey was included from the individual record (IR file) file using standard demographic and health survey datasets of Ethiopia. Using multilevel logistic regression models, we investigated the relationship between several independent factors and women’s independent decision-making not to use contraception. The adjusted odds ratios were evaluated using 95% confidence intervals.

**Results:**

A total of 5,598 currently married women were included in this study. Individual level factors significantly associated with women independent decision making on not to use contraceptive were female-led households (AOR = 2.11; 95% CI = 1.60–2.78), being orthodox ( AOR = 1.84; 95% CI = 1.39–2.44 ) and protestant ( AOR = 1.62; 95% CI = 1.17–2.23), and belonging to more than one union (AOR = 1.48; 95% CI = 1.12–1.95). Whereas, low community education (AOR = 1.19; 95%= 1.00-1.49) and regions: in Tigray (AOR = 2.19; 95%CI = 1.51–3.16), Afar (AOR = 1.74; 95% CI = 1.14–2.64), Amhara (AOR = 2.45; 95% CI = 1.71–3.500), South Nations Nationality (AOR = 1.87; 95% CI = 1.32–2.65), Gambela (AOR = 2.58; 95% CI = 1.73–3.84), Hareri (AOR = 3.93; 95% CI = 2.62–5.88), and Dre DDewa (AOR = 1.66; 95% CI = 1.12–2.45) were community-level factors.

**Conclusion:**

Women’s independent decision-making power not to use contraceptives was low and greatly affected by both individual and community-level factors. Therefore, it is necessary to develop policies and create programs that promote women’s empowerment by incorporating their partners in each region of the nation to encourage women’s independent decision-making authority to use or not to use a contraceptive.

## Background

The number of children the couples want to have changed over time and couples disagreed on fertility preferences or desires [1–4]. Encouraging women’s decision-making to use or not to use contraceptives is documented as an important solution that can change the fertility rate, decrease morbidity and mortality and increase health service utilization [5–8]. However, women often have less power in relationships due to their economic, political and sociocultural status and may not be in a position to protect themselves from gender-based violence, unwanted sexual intercourse, unwanted pregnancy, sexually transmitted infections, depression, to make their own decisions about sex, to equal treatment and to marry or not to marry [9]. Women have minimal autonomy in many cultures, thus it is critical to get (1) a better understanding of the factors influencing their decision-making autonomy; and (2) differences among regions and socio-cultural situations within the same country. Previous research has found that women with less domestic autonomy are less likely to make innovative judgments [10].

Decisions about contraceptive use and childbearing may be confounded by unequal power relations [11]. Where couples disagree on fertility preferences or desires, men’s power in a relationship may contribute to greater influence on whether to use or not to use contraception [12]. Women have been playing a great role, not only in the enhancement of family well-being but also in the progress of the financial, political social, and ecological atmosphere [13]. Family planning use and not use in developing countries are challenged by social and environmental factors that mitigate women’s ability to decide independently and freely [14, 15]. In Ethiopia, men are dominant decision-makers in most family matters, including reproductive health issues [16, 17].

Approximately 14 million unintended pregnancies are recorded annually in Sub-Saharan Africa [18]. Reproductive control including pregnancy coercion (coercion by male partners to become pregnant) 19% and birth control sabotage (partner interference with contraception) 15% are common among young women [19]. In Sub-Saharan Africa, the secret use of contraceptives among women accounts for between 6 and 20% of all contraceptive use [20, 21]. In Saudi Arabia, approximately one-fourth (26.7%) of women reported that their spouses coerced them not to use any contraception [22].

A study conducted in Ethiopia showed that those who do not use modern contraceptives (16.6%) reasoned that it is due to their husband’s dominance [23] and 42.0% of non-users are not involved in a decision it is decided by their husband [24]. Around 42.0% of women said that they have jointly decided not to use a contraceptive method, while a smaller proportion of women had to decide by themselves [22]. Other evidence in Ethiopia showed that because of the male dominance in the culture, women would be forced to bear a large number of children [25].

The Independent decision-making power of women in family planning is defined as a woman’s ability to freely decide individually to use or not to use contraceptives by herself (but not including male family planning utilization ) [17]. Therefore, efforts need to be made for women’s involvement in family planning either to use modern contraceptives or to support each other on when to start and stop the contraceptive and thereby regulate their fertility [17, 26]. Research shows that couples often disagree about the desirability of pregnancy and the use and not to use of contraceptives because mostly the decision is going through the partner [27].

Different shreds of evidence show women’s decision power to use contraceptives in Sub-Saharan African countries including Ethiopia [28, 29]. Nevertheless, to the best of our knowledge, no study has been conducted in Ethiopia that demonstrates women’s independent decision-making power on not to utilize contraception. As a result, the objective of this study was to assess women’s independent decision-making power on not to use contraceptives and determinants among currently married women in Ethiopia.

## Methods

### Data sources

Data from Ethiopia’s demographic and health survey were used in the study ( 2016 EDHS data ). The DHS Program has been collaborating with developing nations all around the world to gather information on important health issues, such as fertility [30]. This typical Demographic and Health Survey is a population-based survey that is nationally representative, contains high-quality data that are gathered using uniform questionnaires, and adheres to defined data gathering techniques. The Federal Ministry of Health (FMoH) and Ethiopia Public Public Health Institute collaborate with the Central Statistical Agency to perform the DHS data in Ethiopia every five years in the nine regional states and two administrative cities. After emailing DHS through personal accounts and providing a justification for the request, data were received from the DHS website (URL: www.dhsprogram.com). After emailing DHS through personal accounts and providing a justification for the request, data were received from the DHS website (URL: www.dhsprogram.com) [30].

The survey target groups were women aged 15–49 and men aged 15–59 in randomly selected households with a multi-stage stratified cluster sampling design. The study involved a cluster sampling process of 443 enumeration areas with 645 clusters. The sample frame usually excludes nomadic and institutional groups such as prisoners and hotel occupants. Detailed information was collected on the background characteristics of the respondents including maternal health and child health [31]. The data for this study were extracted from the individual record (IR file) file. A total of 5,598 unweighted currently married women who are not pregnant and are not current users of contraceptives were included.

### Eligibility identification

Reproductive age group women aged (15–49 years ) currently married women who are not pregnant and are not current users of contraceptives included in this study.

### Variables and operational definitions

The outcome variable for this investigation was “married women’s independent decision-making power on not to use any contraceptive”. For this investigation, the dependent variable was categorized into “no independent decision making = 0” (for married reproductive-age women who reported that the decision-maker not to use any contraceptive method was made by her husband/partner, joint, family members, or others) and “independent decision-making power = 1” (for married reproductive-age group women who reported that the decision-maker on their decision making not to use any contraceptive was made by herself only) [16, 32].

For this study, the independent variables were categorized as individual-level variables (age of the respondents, educational status of the couples, occupational status of the couples, women’s autonomy to health care, head of the household, and living children) and community-level variables which include (residence, community-level education, and regions).

Community education level was categorized into two as low community education level and high education level using the aggregation of the individual women’s education levels of primary, secondary, and higher which can show the overall educational status of women in the cluster based on median level [33].

### Statistical analysis

We use stata version 16 software for data cleansing. The analysis was conducted after sample weight because sample allocation to the different regions as well as urban and rural areas was not proportional.

### Bi-variable and multivariable multilevel logistic regression

The effect of each predictor on the dependent variable was checked. All predictors with a p-value of less than 0.25 in the bivariate multi-level logistic regression analysis were considered as a candidate for multivariable multilevel logistic regression analysis.

### Model building

Model, I (null model), model II, model III, and model IV were fitted for this study. The null model was applied without any independent variables to test random variability in the intercept. Model II and model III was applied for individual level and community level explanatory variable respectively. Model IV applied for both individual-level and community-level explanatory predictors simultaneously. The fitted model was :

$${log}\left(\frac{ {\pi }_{ij}}{1-{\pi }_{ij}}\right)$$+$${\beta }_{0}+{\beta }_{1}{X}_{1ij}+\dots +{\beta }_{n}{X}_{nij}{+e}_{ij}+{\mu }_{oj}$$

   Where ${\pi }_{ij}$indicates the probability of women who had decision making on not to use any contraceptive, $1-{\pi }_{ij}$ is the probability of the women who had no decision-making power on not using any contraceptive methods. ${\beta }_{0}$ is the log odds of the intercept, ${\beta }_{1}\dots {\beta }_{n}$ the amount of effect by the individual and community level variables,${X}_{1}\dots {X}_{n}$ is the independent variable at the individual and community level, ${e}_{ij}$ is the random error at the individual level and ${\mu }_{oj}$ is the random error at the community level. Intra correlation coefficient [(ICC) (variance partition coefficient )] and the percentage change in variance (PCV) were used to estimate the random effect variation of effects).

$$\text{ICC was calculated as ICC}= \left(\frac{The varince of the null model }{\text{t}\text{h}\text{e} \text{v}\text{a}\text{r}\text{i}\text{a}\text{n}\text{c}\text{e} \text{o}\text{f} \text{t}\text{h}\text{e} \text{n}\text{u}\text{l}\text{l} \text{m}\text{o}\text{d}\text{e}\text{l}+\frac{{{\uppi }}^{2}}{3}}\right)$$ and

$\text{PCV was calculated as PCV}=\left(\frac{{V}_{e- }{V}_{mi}}{{V}_{e}}\right)$, where ${V}_{e}$ is the variance of married women who has independent decision-making power on not to use contraceptive in the null model (model-I), ${V}_{mi}$ is the variance in the successive model (model IV or full model). While checking Akaike’s information criteria and Bayesian information criteria for model fitness, the model with the low loglikelihood ratio and AIC value was the best fitted in model IV (full model). To predict the statistically significant effect of variables on women’s independent decision-making power on not to use contraceptives in multivariable multi-level analysis adjust odds ratio with a 95% confidence interval was utilized.

## Results

### Descriptive characteristics

Greater than three fourth 4,352 (77.74%) currently married women and not using contraceptives were found in the age category of 25–49 years. The majority of the respondents 4,729 (80.05%) were living in rural areas. Greater than half 3,268 ( 58.38%) and 3,041 (54.32%) of the respondents were not engaged to work and reaching the health facility was the big problem respectively. About two in five 2,035 (36.35%) women belong to a household having live children greater than four. Greater than half of 3,111 (55.57%) of the respondents were not exposed to the health facility for sick care or any other purpose in the last 12 months ( Table 1).

**Table 1:** Unweighted and weighted percentage distribution of selected characteristics, 2022

Characteristics	unweighted	Weighted	Unweighted Percent
**Maternal age**			
15–19	385	308	6.88
20–24	861	749	15.38
25–49	4352	4307	77.74
**Household head**			
Female	1,260	767	22.51
Male	4338	4597	77.49
**Residence**			
Urban	1,117	635	19.95
Rural	4,481	4,729	80.05
**Maternal occupation**			
No work	3,268	2,949	58.38
Employed	2,330	2,415	41.62
**Religion**			
Orthodox	1,646	1,889	29.40
Moslem	2896	2247	51.73
Protestant	942	1,074	16.83
Catholic/traditional/other	114	154	2.04
**Maternal current working status**			
No work	3,961	3,864	70.76
Has work	1637	1,500	29.24
**Husband education**			
No education	2,998	2,788	53.55
Primary	1,546	1,896	27.62
Secondary	574	405	10.25
Higher	480	275	8.57
**Number of unions**			
Once	4,681	4,403	83.62
More than once	917	961	1638
**Community Education**			
Low	29,11	2,673	52.12
Hight	2,674	2688	47.88
**Living children**			
0	558	418	9.97
1–2	1,532	1,374	27.37
3–4	1,473	1,399	26.31
4+	2,035	2,173	36.35
**They visited a health facility in the last 12 months**			
No	3,111	3,012	55.57
Yes	2,487	2,352	44.43
**Visited by fieldworker in last 12 months**			
No	4,160	3,970	74.31
Yes	1438	1,394	25.69
**Distance to the health facility**			
Big problem	3,041	3,238	54.32
No problem	2557	2126	45.68
**Regions**			
Tigray	537	363	9.59
Afar	640	69	11.43
Amhara	490	1,051	8.75
Oromia	778	2,365	13.9
Somalia	746	242	13.33
Benishangul gumiz	488	69	8.72
South nations and nationaliyt	567	1,017	10.13
Gambela	422	16	7.54
Hareri	320	14	5.72
Addis Abeba	253	129	4.52
Dire Dawa	357	30	6.38
Total	5,598	5,364	100

## Bivariate analysis

Table 2 shows the bivariate relationship between some selected explanatory variables. Current maternal age, household head, maternal occupation, religion, husband education, number union, community education, living children, Visited health facility in last 12 months, visited by fieldworker in the last 12 months and region were with p < 0.25.

**Table 2:** Weighted percentage of selected characteristics and maternal independent decision-making not to use contraceptives in Ethiopia using 2016 EDHS data, 2022

Characteristics	Women’s independent decision-making power to not to use contraceptive		Total	Significant
	**No**	**yes**		
**Maternal age**				
15–19	246	62	308	< 0.001
20–24	573	177	749	
25–49	2904	1403	4307	
**Household head**				
Female	457	310	767	< 0.001
Male	3266	1332	4597	
**Residence**				
Rural	3300	1429	4729	0.09
Urban	422	213	635	
**Maternal occupation**				
No work	2099	850	2949	0.002
Employed	1624	792	2415	
**Religion**				
Orthodox	1180	708	1889	< 0.001
Moslem	1689	558	2248	
Protestant	750	324	1074	
catholic/traditional/other	103	52	154	
**Maternal current working status**				
No worke	2703	1161	3864	0.09
Worke	2703	481	1500	
**Husband education**				
No education	1895	894	2788	0.012
Primary	1348	548	1896	
Secondary	272	132	405	
Higher	208	68	276	
**Number of unions**				
Once	3155	1248	4403	< 0.001
More than once	567	394	961	
**Community Education**				
Low	1819	855	2673	0.03
Hight	1901	787	2688	
**Living children**				
0	304	114	418	< 0.001
1–2	1014	360	1374	
3–4	939	460	1399	
4+	1466	708	2173	
**They Visited health facility in the last 12 months**				
No	2140	872	3012	0.003
Yes	1582	770	2352	
**Visited by fieldworker in last 12 months**				
No	2737	1233	3970	0.20
Yes	986	409	1394	
**Distance to the health facility**				
Big problem	2285	953	3238	0.08
No problem	1437	689	2126	
**Regions**				
Tigray	230	133	363	< 0.001
Afar	46	23	69	
Amhara	632	419	1051	
Oromia	1770	595	2365	
Somalia	186	56	242	
Benishangul gumiz	53	16	69	
South nations and nationality	681	336	1017	
Gambela	9	7	16	
Hareri	7	7	14	
Addis Abeba	88	41	129	
Dire dawa	20	10	30	

### Multivariate multilevel analysis

In multivariate multilevel analyis, the individual level factors: house hold lead by female (AOR = 2.11; 95% CI = 1.60–2.78), religion being orthodox ( AOR = 1.84; 95% CI = 1.39–2.44 ) and protestant ( AOR = 1.62; 95% CI = 1.17–2.23), and more than one union (AOR = 1.48; 95% CI = 1.12–1.95) were significantly associated with women independent decision making on not to use contraceptive. Where as, at community level low community education (AOR = 1.19; 95%= 1.00-1.49) and regions: in tigiray (AOR = 2.19; 95%CI = 1.51–3.16), Afar (AOR = 1.74; 95% CI = 1.14–2.64), Amhara (AOR = 2.45; 95% CI = 1.71–3.500), South Nations Nationality (AOR = 1.87; 95% CI = 1.32–2.65), Gambela (AOR = 2.58; 95% CI = 1.73–3.84), Hareri (AOR = 3.93; 95% CI = 2.62–5.88), and Dre dawa (AOR = 1.66; 95% CI = 1.12–2.45) were significantly associated with women independent decision making on not to use contraceptive.

In the full model (model IV ) which includes both individual and community level factors: house hold lead by female (AOR = 2.10; 95% CI 1.59–2.78), religion being orthodox ( AOR = 1.52; 95%CI = 1.05–2.20), protestant ( AOR = 1.50; 95% CI = 1.01–2.23) and catholic/traditional/other (AOR = 1.8; 95% CI = 0.72–4.51), more than one union (AOR = 1.43; 95% CI = 1.07–1.43), in regions: Tigiray (AOR = 1.66; 95% CI = 1.00-2.82), in Afar (AOR = 1.72; 95% CI = 1.11–2.67), Amhara region (AOR = 1.89; 95% CI = 1.13–3.16), South Nations Nationality (AOR = 1.47; 95% CI = 0.92–2.37), in Gambela (AOR = 2.06; 95%CI = 1.20–3.54), Hareri (AOR = 4.86; 95% CI = 3.10–7.61), and in Dre dawa city administration (AOR = 1.72; 95% CI = 1.12–2.63) were significantly associated with women’s independent decision-making power on not to use contraceptive (Table 3).

**Table 3:** Multivariable multilevel logistic regression of individual and community level variables associated with independent women decision making not to use contraceptives in Ethiopia using EDHS data, 2022

Characteristics	Independent women decision making not to use contraceptives ( adjust analysis (AOR, 95%CI)		
	**Model II**	**Model III**	**Model IV**
**Household head**			
Female	2.11(1.60–2.78)*		2.10 (1.59–2.78)*
Male	1	----	1
**Religion**			
Orthodox	1.84 (1.39–2.44)*		1.52 (1.05–2.20)*
Moslem	1	-----	1
Protestant	1.62 (1.17–2.23)*		1.50 (1.01–2.23) *
Catholic/traditional/other	1.86(0.74–4.69)		1.81 (0.72–4.51) *
**Number of unions**			
Once	1	---	1
More than once	1.48 (1.12–1.95)*		1.43 (1.07–1.43) *
**Community Education**			
Low		1.19 (1.00-1.49)*	1.16 (0.93–1.44)
High		1	
**Region**			
Tigray		2.19 (1.51–3.16)*	1.66 (1.00-2.82)*
Afar		1.74 (1.14–2.64)*	1.72 (1.11–2.67) *
Amhara		2.45 (1.71–3.500)*	1.89 (1.13–3.16)*
Oromia		1.08 (0.73–1.61)	1.02 (0.65–1.61)
Somalia	---	1	1
Benishangul Gumiz		0.99 (0.66–1.48)	0.89 (0.56–1.43)
South Nations and Nationality		1.87 (1.32–2.65) *	1.47 (0.92–2.37) *
Gambela		2.58 (1.73–3.84)*	2.06 (1.20–3.54)*
Hariri		3.93 (2.62–5.88) *	4.86 (3.10–7.61)*
Addis Abeba		1.49 (0.95–2.34)	1.29 (0.74–2.24)
Dire Dawa		1.66 (1.12–2.45)*	1.72 (1.12–2.63)*
**Model fitness**			
Log-likelihood	-3026.313	-3082.415	-3012.847
AIC	6092.625	6192.829	6089.694
BIC	6225.229	6285.609	6301.762

Note: AOR; Adjusted Odds Ratio, AIC; Akaike’s Information Criterion, BIC; Bayesian Information Criterion, * *p*-value less than 0.05

In terms of model fitness, the complete model (Model IV), which include all individual and community level factors had the lowest log-likelihood ratio (-3012.847) and lowest AIC (6089.694) and were thus considered the best fit model for predicting women’s independent decision making to not use contraceptive among married women (Table 3).

## Discussions

One of the most important aspects of women’s sexual and reproductive health rights is their autonomy in making decisions about whether or not to use contraception. [34]. A significant portion of women who do not want to get pregnant do not use contraception in resource-constrained nations, and as a result, those who do not want to use contraceptives for a variety of reasons use family planning methods that are enforced by their husbands, families, or others. In contrast, most partners in developing nations accord women a lower status in all areas of decision-making [35]. Most of the time, her spouse or family decides whether or not she should use family planning methods (whether due to a preference for fertility, a concern about the adverse effects of family planning methods, or for any other reason). So this why this study was conducted?

In this study, among women who want to not use contraceptives, only 1,754 (31.33%) of them were decided by women independently. The remaining 3, 256 (58.16%) and 588 (10.50%) were decided by joint and husband/partner respectively. Similar findings from the neighboring country showed that the contraceptive is much more likely to be used when the husband rather than the wife wants to cease childbearing [36]. However, mostly, women’s contraceptive utilization and not use of any family planning methods is not merely the responsibility of women; yet, general approval was done by spousal interest [37].

Consequently, Becker [37, 38] already stated that there are circumstances in which the calculated met need and unmet need for contraceptive is meaningfully dissimilar for partner and wife, which means that the husband’s influence is much superior. This suggested that due to different reasons including cultural values, there are unexpressed feelings that men have more absolute independent decision-making power regarding contraceptive use and not to use than their wives, even though from a medical viewpoint, most family planning methods are developed for women, and family planning services have principally been provided to individual women too, not to couples or partner [6, 9, 12, 35, 39]. The preceding shreds of evidence indeed indicated that the husband’s fertility intention has an influence on the wife’s use and not use of contraceptive methods [40, 41].

These data demonstrated that women’s independent decision-making power on not to use contraceptives was significantly associated with women heading households compared to males headed households and this result was in line with previous evidence which stated that women heading households were found to have higher odds of contraceptive decisions making including discontinuation compared to males headed households, [42]. This might be the fact that leading the household may increase the participation of women in social issues and may increase their participation in household decision-making (purchasing of large household materials), and may have also good interaction with their neighbors and families. Inadation they have a chance to visit health institutions and well discussed with health professionals about information that could be accurate, misconceptions rumors, and myths [43]. So that if the women gate this kind of opportunity, they will have the power of independent decision making to use or not to use contraceptives.

Concerning religion, remarkable discoveries had investigated that one’s religious affiliation may have some effects on the women’s independent decision-making power regarding reproductive health rights. Thus, this study also agreed that orthodox, protestant, and catholic/traditional/others were more likely to make an independent decision making not to use contraceptives compared to the Muslim religion and this investigation is in line with earlier studies [44, 45].

The reason could be because according to Islamic culture, women are expected to respect men and not challenge their authority. They are expected to be submissive under the control of males. This may help to explain why Muslim women are less inclined to make decisions regarding their reproductive health.

We found that, compared to women in the Somalia region, women in Tigray, Afar, Amhara, South National Nationality, Gambella, Hariri, and Dire Dawa were more likely to decide independently to not use contraceptives. This is in line with a previous study conducted in Ghana [46] which showed regional differences. This is because Ethiopia has a diversity of cultures and which protects women’s rights to exercise their power, also in some regions of Ethiopia there are governmental and non-governmental organizations that work on women empowerment ( like the no behind women project) and some of the regions are small peripheral. This may be the reason why there are regional variations in women’s independent decision-making power on not to use contraceptives.

Another finding from our study that constantly worth noting is that women experiencing more than one union had more likely to decide independently to not use contraceptives. This is in line with a study done in [47] which stated that marital suspicion had a 26% reduced relative risk of jointly deciding whether or not to use contraception when compared with women who had not experienced marital suspicion. This confirmed that the partner or husband is jealous, accusing the respondent of being unfaithful or insisting on knowing where the respondent is at all times. In addition, if she has more than one union, she develops suspicion of her partner, then she exercises her reproductive right independently [47].

## Conclusion

Women’s independent decision-making not to use contraceptives is greatly affected by both individual and community-level characteristics. Therefore, Ethiopian programmers and policy initiatives including non-governmental organizations must develop policies and create potent programming agents that boost women’s independent decision-making power to support women’s autonomy in deciding whether or not to take a contraceptive. Furthermore, in order to achieve the subsequent Sustainable Development Goals, region- and culture-based interventions that incorporate male involvement tactics are necessary because Ethiopia is home to multiethnic cultures.

## Limitation

Self-reported responses to the events that occurred in the past may be prejudiced by recall bias. Moreover, information about women’s independent decision-making power in family planning was collected based on self-reporting, which is likely to be exposed to social desirability bias due to its socio-cultural values.

Since the study considered currently married women who are not pregnant and are not currently using the family planning methods, There may be threats of internal and external validity because some of the observations were dropped during data cleaning and the study didn’t consider pregnant women. Hence, the study may be suffered from selection bias. Generally, carefulness is required while summarizing this study since it focused on only currently married women who are not pregnant and are not current users of contraceptive methods.

## Data Availability

All the data sets are available on the hand of the corresponding author.

## Abbreviations

DHS: Demographic and Health Survey
EDHS: Ethiopian Demographic and Health Survey
SSA: Sub-Saharan Africa
WHO: World Health Organization
